# Association between Smoking Status and Obesity in a Nationwide Survey of Japanese Adults

**DOI:** 10.1371/journal.pone.0148926

**Published:** 2016-03-23

**Authors:** Taku Watanabe, Ichizo Tsujino, Satoshi Konno, Yoichi M. Ito, Chisa Takashina, Takahiro Sato, Akira Isada, Hiroshi Ohira, Yoshinori Ohtsuka, Yuma Fukutomi, Hiroyuki Nakamura, Yukio Kawagishi, Chiharu Okada, Nobuyuki Hizawa, Masami Taniguchi, Akira Akasawa, Masaharu Nishimura

**Affiliations:** 1 First Department of Medicine, Hokkaido University School of Medicine, Sapporo, Hokkaido, Japan; 2 Department of Biostatistics, Graduate School of Medicine, Hokkaido University, Sapporo, Hokkaido, Japan; 3 Clinical Research Center for Allergy and Rheumatology, Sagamihara National Hospital, Sagamihara, Kanagawa, Japan; 4 Department of Environmental and Preventive Medicine, Graduate School of Medical Science, Kanazawa University, Kanazawa, Japan; 5 Department of Internal Medicine, Kurobe City Hospital, Kurobe, Toyama, Japan; 6 Department of Medicine, Headquarter of National Hospital Organization, Tokyo, Japan; 7 Department of Allergy, National Hospital Organization Tokyo Medical Center, Tokyo, Japan; 8 Department of Pulmonary Medicine, Faculty of Medicine, University of Tsukuba, Tsukuba, Ibaraki, Japan; 9 Department of Allergy, Tokyo Metropolitan Children’s Medical Center, Tokyo, Japan; West Virginia University, UNITED STATES

## Abstract

**Objective:**

A positive association between the number of cigarettes smoked per day and obesity has been reported, whereas how other smoking-related indices, such as pack-years and duration of smoking, are related with obesity has been less investigated. We analyzed the age-adjusted cross-sectional association between smoking and obesity in a general Japanese population.

**Methods:**

We used data from a nationwide epidemiological study of Japanese adults (N = 23,106). We compared the prevalence of obesity (defined as body mass index ≥ 25kg/m^2^) among groups classified by smoking behavior, pack-years, number of cigarettes per day, duration of smoking, and duration and time of smoking cessation.

**Results:**

In men, current smokers had a lower odds ratio (OR) for obesity of 0.80 (95% confidence interval (CI): 0.72–0.88) compared to non-smokers, whereas past smokers had a higher OR of 1.23 (95% CI: 1.09–1.37) compared to current smokers. In women, there were no differences in obesity between the three groups classified by smoking behavior. However, in both sexes, the prevalence of obesity tended to increase with pack-years and the number of cigarettes per day, but not with duration of smoking in current and past smokers. Further, in male smokers, the risks for obesity were markedly higher in short-term heavy smokers compared with long-term light smokers, even with the same number of pack-years. Regarding the impact of smoking cessation, female past smokers who quit smoking at an age > 55-years had an elevated OR of 1.60 (95% CI:1.05–2.38) for obesity.

**Conclusions:**

In a general Japanese population, obesity is progressively associated with pack-years and number of cigarettes per day, but not with the duration of smoking. When investigating the association between obesity and cigarette smoking, the daily smoking burden and the duration of smoking require to be independently considered.

## Introduction

Cigarette smoking is one of the leading causes of preventable morbidity and mortality [1,2]. Several studies have indicated that smoking behavior is closely related to body weight and obesity [3–5], and shown that body weight of past smokers was heavier than that of never smokers [6–8]. The mechanisms underlying the impact of smoking on weight include varying energy intake, physical activity, metabolic rate, and inflammatory status linked to smoking status [5].

Importantly, associations between smoking and obesity have been extensively investigated in large-scale studies of subjects of Western ethnicities, but not in Asian populations [9–11]. This is of clinical importance because obesity-related complications, such as diabetes and metabolic syndrome reportedly occur at a lower BMI in Asian subjects compared with Western subjects [12–15]. Indeed, although obesity is now commonly defined in adults as a BMI ≥ 30 kg/m^2^ [16], the World Health Organization (WHO) western pacific regions have provided a different cut-off level for obesity in Asians (BMI ≥ 25 kg/m^2^) [17]. Likewise, the Japan Society for the Study of Obesity has defined obesity as BMI ≥ 25 kg/m^2^ for Japanese subjects [18]. In addition to different definitions of obesity used for different populations, a recent report noted that smoking prevalence significantly varied in Asian countries, including Japan [19]. These findings indicate the need for dedicated studies on the association between smoking and obesity in Asian countries.

In the prior publications on obesity, the number of cigarettes per day (CPD) rather than pack-years has been used. Importantly, pack-years have two elements: the degree of daily cigarette smoking (pack/day) and duration of smoking (years). However, few studies have focused separately on the impact of the two elements of pack-years on obesity. Regarding the impact of smoking cessation on body weight, some previous studies have examined the association between the duration of smoking cessation and body weight [5]. To date, however, few studies have examined the impact of age at quitting smoking on body weight [8].

The aim of this study was to analyze the association of BMI and the prevalence of obesity with the following smoking-related factors: (1) smoking status (current, past, or never smoker), (2) pack-years, (3) the number of CPD, (4) duration of smoking, (5) both the number of CPD and duration of smoking, (6) the number of years since quitting smoking, and (7) the age at time of smoking cessation in past smokers.

## Methods

### Study design and participants

Our data were drawn from a population-based, cross-sectional observational study conducted from July 2006 to February 2007 on Japanese subjects 20–79 years of age, living in ten different areas of Japan, to determine the nationwide prevalence of asthma [20]. Detailed methods for selecting areas, participants, questionnaire distribution, and response rates in each area have been described previously [20]. In brief, subjects were asked to complete the Japanese version of the European Community Respiratory Health Survey questionnaire [21], which included questions on height, weight and smoking status. We classified subjects according to their smoking habits [20]. The overall response rate of this study was 70.6% [20], and after excluding subjects who did not complete questions on height, weight, and CPD, data from 23,106 subjects (69.4% of initial participants of the survey; 11,245 males and 11,861 females) were analyzed.

### Statistical analysis

#### Comparison of BMI/prevalence of obesity by smoking status

We compared BMI and prevalence of obesity among current, past, and never smokers using one-way analysis of variance (ANOVA) followed by Tukey-Kramer’s post-hoc test in both sexes. Obesity was defined as BMI ≥ 25 kg/m^2^, as recommended by the WHO western pacific regions [17] and the Japan Society for the Study of Obesity [18]. The prevalence of obesity was calculated by dividing the number of obese subjects by the number of total subjects in each smoking status group.

#### Association between age and prevalence of obesity in groups with different by smoking status

Because age is known to have a critical impact on obesity [22–24], we evaluated the relationship between age and the prevalence of obesity for each smoking status group. We used the second order logistic regression model with intercept, linear, and quadratic terms; logit(P) = intercept +αAge + β(Age-mean Age)^2^.

#### Age-adjusted comparisons of BMI and odds ratio (OR) for obesity in groups classified by smoking status

Overall age-adjusted BMI (10-year categories) was compared among the three smoking status groups using ANOVA followed by Tukey-Kramer’s post-hoc test in men and women. Odds ratios (OR) for obesity and 95% confidence intervals (CI) were calculated by multiple logistic regression model using the data of never and current-smokers as reference values. The factor used for adjustment was age categories (20–29, 30–39, 40–49, 50–59, 60–69, 70–79 years) [20].

#### Age-adjusted association between indices of smoking burden (pack-years, CPD, and duration of smoking) and obesity

The prevalence of obesity was first analyzed by Cochran-Mantel-Haenszel (CMH) trend test [25] after adjustment for age among current/past smokers stratified by pack-years (≤ 10, 11–20, 21–30, and >30). Age-adjusted OR was calculated as above using the data of never smokers as the reference. Subjects were then classified according to the two elements of pack-years: CPD (Men; ≤ 10, 11–20, 21–30, 30–40, and >40, Women; ≤ 10, 11–20, and >20) and duration of smoking (<10 years, 10–19 years, 20–34 years, and ≥35 years), and were analyzed in the same way as pack-years.

Age-adjusted ORs for obesity was also compared among groups stratified by both CPD and duration of smoking in male smokers. In this analysis, CPD was re-stratified by ≤ 10, 11–20, and >20, and duration of smoking was re-stratified by <10 years, 10–34 years, and ≥35 years.

#### Association between the duration of smoking cessation/the age at quitting smoking and obesity in past smokers

Past smokers were categorized by duration of smoking cessation and the age of smoking cessation. Then the prevalence of obesity was analyzed by CMH trend test adjusted for age. Age-adjusted ORs for obesity were compared among groups. Furthermore, past smokers with a cigarette consumption of more than 20 cigarettes per day were defined as heavy smokers, and we performed a sub-analysis to examine the relationship between obesity and duration of smoking cessation in heavy smokers or non-heavy smokers.

Statistical analyses were performed using JMP^®^ Pro for Windows, version 10.0 (SAS Institute Inc., Cary, NC, USA). For all statistical analyses, P < 0.05 was considered statistically significant. This study was approved by the Ethics Committee of Sagamihara National Hospital and Hokkaido University. Informed consent was obtained from all the patients.

## Results

### Comparison of BMI/prevalence of obesity by smoking status

Table 1 shows general characteristics of subjects. Of the entire 23,106 participants, 11,245 (48.7%) were men and 11,861 (51.3%) were women. In both sexes, BMI and the prevalence of obesity of current-smokers were significantly lower than those of the never smokers. Conversely, BMI of past-smokers was significantly higher than that of current-smokers. There were also significant differences in the mean age among the three smoking statuses in both sexes.

**Table 1:** Characteristics of the Groups Classified by Smoking Status.

	Smoking Status			
**Men**	**Overall**	**Never**	**Current**	**Past**
**n (%)**	11 245 (100.0)	3 883 (34.5)	4 732 (42.1)	2 630 (23.4)
**BMI (kg/m**^**2**^**)**	23.2 ± 3.3	23.3 ± 3.3	23.0 ± 3.3*	23.4 ± 3.1†
**Prevalence of obesity(%)**	24.9	25.9	23.3‡	26.6§
**Age (year)**	50.3 ± 16.3	49.4 ± 17.8	47.3 ± 15.1*	57.1 ± 14.1† ‡
**Pack-years**	**-**	**-**	30.2 ± 23.0	31.7 ± 26.2§
**Cigarettes per day**	**-**	**-**	20.4 ± 10.1	23.0 ± 13.2†
**Women**	**Overall**	**Never**	**Current**	**Past**
**n (%)**	11 861 (100.0)	9 907 (83.5)	1 324 (11.1)	630 (5.3)
**BMI (kg/m**^**2**^**)**	22.1 ± 3.4	22.1 ± 3.4	21.5 ± 3.3*	22.0 ± 3.3†
**Prevalence of obesity(%)**	16.7	17	13.9‡	17.3
**Age (year)**	50.8 ± 16.0	52.0 ± 16.0	43.8 ± 13.9*	47.3 ± 14.9† ‡
**Pack-years**	**-**	**-**	16.5 ± 13.5	13.0 ± 16.6†
**Cigarettes per day**	**-**	**-**	14.0 ± 7.4	13.3 ± 8.9

*p<0.01 v.s. never-smokers

^†^p<0.01 v.s. current-smokers

^‡^p<0.05 v.s. never-smokers

^§^p<0.05 v.s. current-smokers

### Association between age and prevalence of obesity in groups with different by smoking status

**Fig 1:**
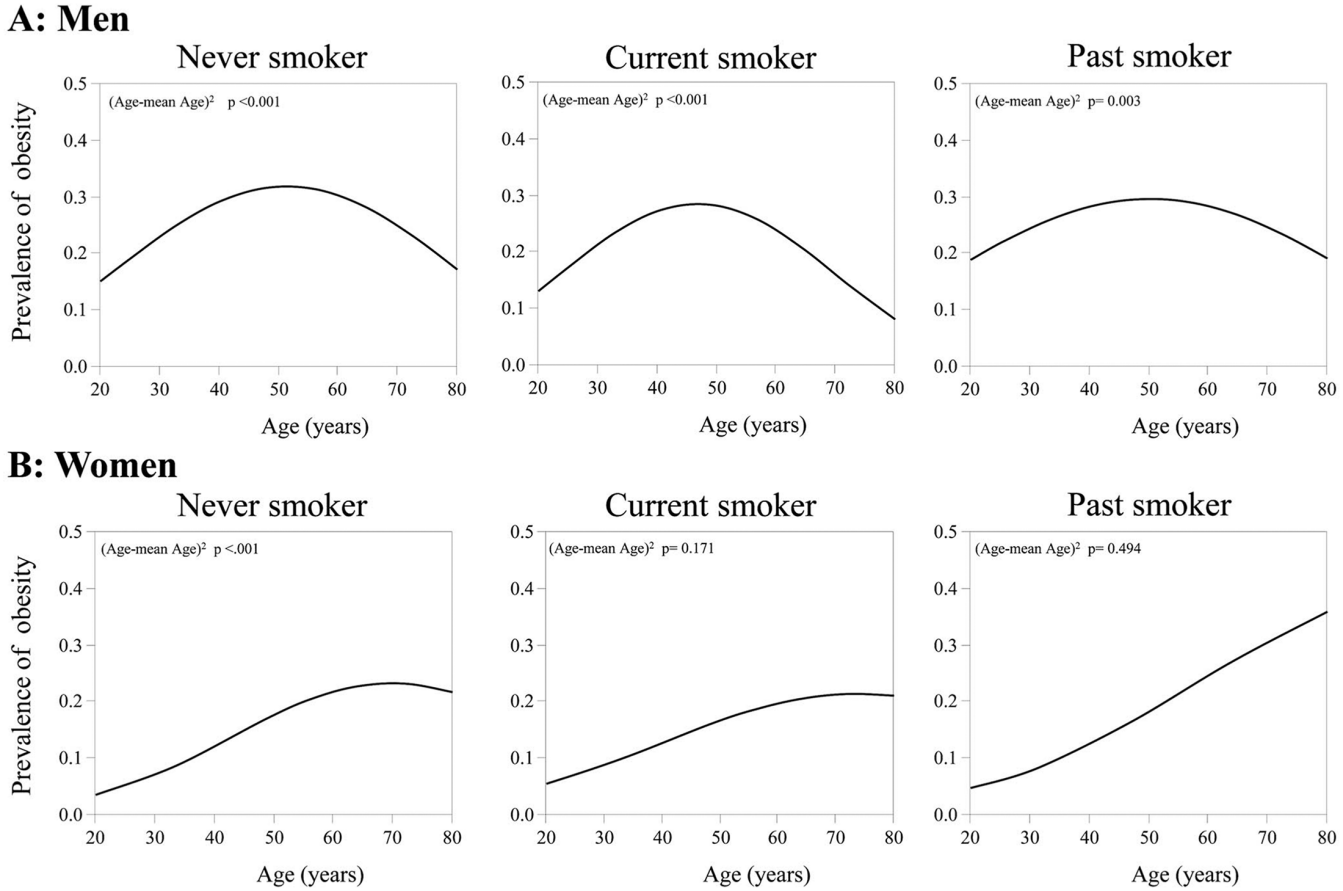
Association between age and the prevalence of obesity by smoking status in men and women. Data were determined using the second order logistic regression model with intercept, linear, and quadratic terms: logit(P) = intercept +αAge +β(Age-mean Age)2.

The association between age and the prevalence of obesity were different among smoking status groups in men and women (Fig 1). In men, the association showed a non-linear quadratic trend and the prevalence of obesity peaked at around age 50 irrespective of smoking status. In contrast, in female never smokers, the association showed a quadratic trend, whereas the prevalence of obesity peaked at about 70 years. Furthermore, in past and current female smokers, the association was somewhat linear.

### Age-adjusted comparisons of BMI and odds ratio (OR) for obesity in groups classified by smoking status

Table 2 shows that, in men, current smokers had lower age-adjusted BMI and Table 3 shows ORs for obesity compared to both never or past smokers, while there were no such differences between past smokers and never smokers. In women, age-adjusted BMI and ORs did not differ among the three smoking status groups.

**Table 2:** Comparison of age-adjusted mean body mass index among never, current, and past smokers.

	Smoking Status		
	Never	Current	Past
**Men**	23.3	23.0*	23.4†
**Women**	22.1	21.9	22.2

*p<0.01 v.s. never-smokers

^†^p<0.01 v.s. current-smokers

**Table 3:** Comparison of age-adjusted overall odds ratio for obesity among never, current, and past smokers.

**Men**		**OR v.s. never**	**95% CI**	**P value**
	**Current**	0.8	0.72–0.88	< 0.001
	**Past**	0.97	0.87–1.09	0.65
		**OR v.s. current**	**95% CI**	**P value**
	**Past**	1.23	1.09–1.37	< 0.001
**Women**		**OR v.s. never**	**95% CI**	**P value**
	**Current**	1.01	0.85–1.19	0.92
	**Past**	1.19	0.95–1.47	0.12
		**OR v.s. current**	**95% CI**	**P value**
	**Past**	1.18	0.90–1.53	0.22

### Age-adjusted association between indices of smoking burden (pack-years, CPD, and duration of smoking) and obesity

In both sexes, there were trends showing that the higher pack-years were, the higher the prevalence of obesity was in both current and past smokers (Tables 4 and 5). In line with this, male current smokers with ≤ 60 pack-years and male past smokers with ≤ 10 pack-years had significantly reduced ORs for obesity and, conversely, male past smokers with > 60 pack-years had a significantly increased OR (adjusted OR 1.55, 95% CI 1.20–1.99, p < 0.001) (Table 4). Similarly, female past smokers with > 30 pack-years had an increased OR (adjusted OR 1.92, 95% CI 1.12–3.20, p = 0.02) (Table 5).

**Table 4:** Comparison of prevalence of obesity and odds ratio for obesity in male smokers stratified by pack-years.

				Cochran-Mantel-Haenszel trend test		Multiple logistic regression		
Smoking status	Pack-years	n	BMI	Prevalence of obesity (%)	P value†	Odds ratio*	95% CI	P value
**Current**	≤ 10	866	22.4±3.4	19.1	<0.001	0.77	0.63–0.94	0.01
	11–20	956	22.9±3.4	21.9		0.68	0.57–0.81	< 0.001
	21–30	854	23.1±3.1	24.7		0.78	0.65–0.93	0.006
	31–60	1551	23.2±3.2	23.9		0.81	0.70–0.93	0.004
	60 <	475	23.4±3.4	28		1.05	0.84–1.30	0.67
**Past**	≤ 10	491	23.1±3.0	23.1	<0.001	0.79	0.63–0.98	0.03
	11–20	513	23.4±3.0	25.5		0.86	0.70–1.07	0.17
	21–30	449	23.5±3.2	26.3		0.96	0.76–1.20	0.71
	31–60	817	23.4±3.0	27.1		1.08	0.90–1.28	0.41
	60 <	315	23.7±3.3	34		1.55	1.20–1.99	< 0.001

*Prevalence of obesity of never smokers was used as the reference value.

BMI, body mass index

^†^Each P value was calculated by Cochran-Mantel-Haenszel trend test among groups stratified by pack-years in current or past smokers.

**Table 5:** Comparison of prevalence of obesity and odds ratio for obesity in female smokers stratified by pack-years.

				Cochran-Mantel-Haenszel trend test		Multiple logistic regression		
Smoking status	Pack-years	n	BMI	Prevalence of obesity (%)	P value†	Odds ratio*	95% CI	P value
**Current**	≤ 10	533	21.1±3.2	10.1	0.009	1.02	0.75–1.36	0.89
	11–20	381	21.2±3.0	11		0.73	0.52–1.00	0.049
	21–30	225	22.2±3.4	19.6		1.15	0.81–1.60	0.42
	30 <	176	22.7±3.8	24.4		1.3	0.91–1.83	0.15
**Past**	≤ 10	379	21.5±3.0	12.1	0.02	0.93	0.67–1.27	0.66
	11–20	111	22.4±3.4	19.8		1.24	0.75–1.95	0.39
	21–30	56	22.8±3.5	26.8		1.52	0.81–2.70	0.19
	30 <	64	23.8±3.9	34.4		1.92	1.12–3.20	0.02

*Prevalence of obesity of never smokers was used as the reference value.

BMI, body mass index

^†^Each P value was calculated by Cochran-Mantel-Haenszel trend test among groups stratified by pack-years in current or past smokers.

Tables 6 and 7 show the results of age-adjusted analysis between CPD and obesity. In men, both current and past smokers exhibited a trend that the higher CPD was, the higher the prevalence of obesity was. In line with this finding, current smokers with ≤ 20 CPD and past smokers with ≤ 10 CPD had significantly decreased ORs and, conversely, the OR of current and past smokers with > 40 CPD had was significantly increased ORs (adjusted OR of current smokers 1.56, 95% CI 1.01–2.39, p = 0.047 and adjusted OR of past smokers 1.76, 95% CI 1.25–2.46, p = 0.001)(Table 6). In women, no such trend was evident in current smokers whereas, in past smokers, the OR was significantly increased in subjects with > 20 CPD (adjusted OR 2.05, 95% CI 1.04–3.85, p = 0.039)(Table 7).

**Table 6:** Comparison of prevalence of obesity and odds ratio for obesity in male smokers stratified by cigarettes smoked per day (CPD).

				Cochran-Mantel-Haenszel trend test		Multiple logistic regression		
Smoking status	CPD	n	BMI	Prevalence of obesity (%)	P value†	Odds ratio*	95% CI	P value
**Current**	≤ 10	1002	22.5±3.2	19.4	<0.001	0.68	0.57–0.81	< 0.001
	11–20	2540	22.9±3.2	22.1		0.75	0.66–0.84	< 0.001
	21–30	724	23.3±3.3	26.5		0.91	0.76–1.09	0.32
	31–40	375	23.6±3.8	30.7		1.09	0.86–1.38	0.47
	40 <	91	24.5±3.9	38.5		1.56	1.01–2.39	0.047
**Past**	≤ 10	495	23.0±3.1	22.4	<0.001	0.78	0.62–0.98	0.031
	11–20	1322	23.3±2.9	25		0.9	0.78–1.04	0.15
	21–30	379	23.6±3.0	28.8		1.1	0.87–1.39	0.42
	31–40	283	24.0±3.5	31.8		1.26	0.97–1.64	0.09
	40 <	152	24.1±3.0	38.8		1.76	1.25–2.46	0.001

*Prevalence of obesity of never smokers was used as the reference value.

BMI, body mass index

^†^Each P value was calculated by Cochran-Mantel-Haenszel trend test among groups stratified by CPD in current or past smokers.

**Table 7:** Comparison of prevalence of obesity and odds ratio for obesity in female smokers stratified by cigarettes smoked per day (CPD).

				Cochran-Mantel-Haenszel trend test		Multiple logistic regression		
Smoking status	CPD	n	BMI	Prevalence of obesity (%)	P value†	Odds ratio*	95% CI	P value
**Current**	≤ 10	645	21.4±3.2	12.1	0.08	0.9	0.70–1.15	0.42
	11–20	608	21.5±3.3	14.8		1.04	0.82–1.31	0.73
	20 <	71	23.0±3.9	22.5		1.61	0.88–2.78	0.12
**Past**	≤ 10	372	21.6±3.1	14.5	0.07	1.02	0.75–1.37	0.89
	11–20	214	22.4±3.4	19.2		1.29	0.90–1.82	0.16
	20 <	44	23.4±4.0	31.8		2.05	1.04–3.85	0.039

*Prevalence of obesity of never smokers was used as the reference value.

BMI, body mass index

^†^Each P value was calculated by Cochran-Mantel-Haenszel trend test among groups stratified by CPD in current or past smokers.

Tables 8 and 9 show results of the age-adjusted analysis between duration of smoking and obesity. There were no specific trends by smoking status in both sexes. However, in men, current smokers who smoked for more than 35 years had a significantly decreased OR (adjusted OR 0.68, 95% CI 0.59–0.79, p < 0.001) compared with never smokers (Table 8). In contrast, in women, current smokers who smoked for less than 10 years had a significantly increased OR (adjusted OR 1.69, 95% CI 1.05–2.62, p = 0.03) compared with never smokers (Table 9).

**Table 8:** Comparison of prevalence of obesity and odds ratio for obesity in male smokers stratified by duration of smoking.

				Cochran-Mantel-Haenszel trend test		Multiple logistic regression		
Smoking status	Duration(year)	n	BMI	Prevalence of obesity (%)	P value†	Odds ratio*	95% CI	P value
**Current**	< 10	593	22.1±3.6	16.9	0.16	0.95	0.72–1.23	0.67
	10–19	980	23.2±3.5	24.6		0.82	0.68–0.99	0.04
	20–34	1432	23.5±3.3	28.1		0.91	0.78–1.05	0.2
	35≤	1696	22.7±3.0	20.4		0.68	0.59–0.79	< 0.001
**Past**	< 10	383	23.2±3.0	23	0.49	0.82	0.63–1.05	0.12
	10–19	592	23.7±3.1	28.6		0.99	0.82–1.20	0.94
	20–34	868	23.7±3.1	29.3		1.07	0.90–1.26	0.44
	35≤	741	22.9±3.0	24.2		0.93	0.76–1.12	0.44

*Prevalence of obesity of never smokers was used as the reference value.

BMI, body mass index

^†^Each P value was calculated by Cochran-Mantel-Haenszel trend test among groups stratified by duration of smoking in current or past smokers

**Table 9:** Comparison of prevalence of obesity and odds ratio for obesity in female smokers stratified by duration of smoking.

				Cochran-Mantel-Haenszel trend test		Multiple logistic regression		
Smoking status	Duration(year)	n	BMI	Prevalence of obesity (%)	P value†	Odds ratio*	95% CI	P value
**Current**	< 10	228	20.9±3.3	11	0.16	1.69	1.05–2.62	0.03
	10–19	396	20.9±3.0	9.6		0.9	0.62–1.27	0.57
	20–34	486	22.0±3.2	16		0.98	0.75–1.25	0.85
	35≤	202	22.3±3.7	20.8		0.94	0.66–1.31	0.72
**Past**	< 10	229	21.2±3.0	11.8	0.49	1.07	0.69–1.60	0.75
	10–19	181	22.0±3.3	14.9		1.04	0.67–1.55	0.86
	20–34	148	22.8±3.4	25		1.4	0.95–2.03	0.09
	35≤	52	23.2±3.8	26.9		1.26	0.66–2.29	0.46

*Prevalence of obesity of never smokers was used as the reference value.

BMI, body mass index

^†^Each P value was calculated by Cochran-Mantel-Haenszel trend test among groups stratified by duration of smoking in current or past smokers

**Fig 2:**
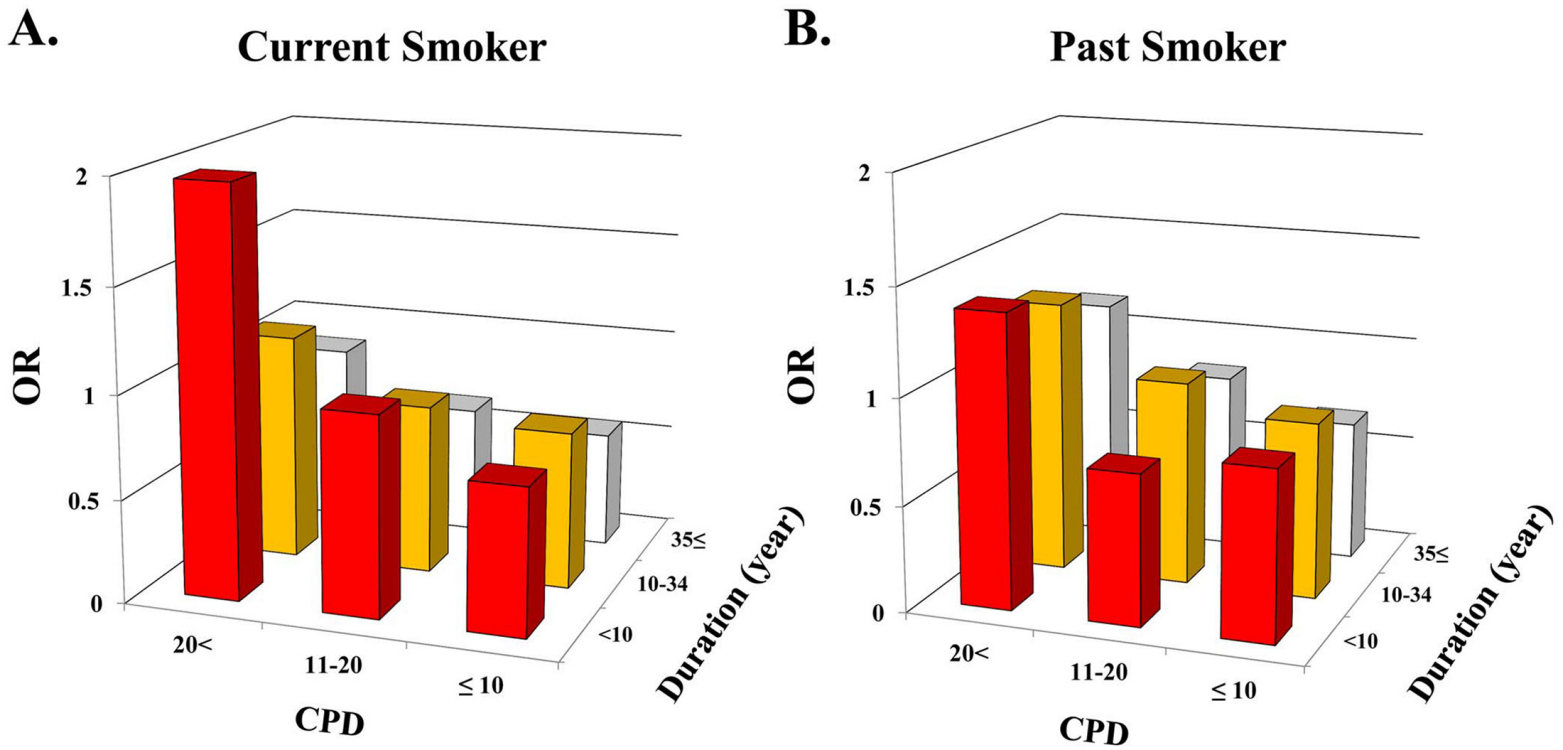
Age-adjusted odds ratios (OR) for obesity of the male smokers by the number of cigarette per day (CPD) and the duration of smoking. Age-adjusted ORs for obesity of the categories stratified by both CPD and duration of smoking in male current smokers and male past smokers. CPD was stratified by ≤ 10, 11–20, and >20. Duration of smoking was stratified by <10 years, 10–34 years, and ≥35 years.

Fig 2 shows the age-adjusted ORs for obesity stratified by both CPD and duration of smoking in male current and past smokers. Male current and past smokers who smoked > 20 CPD for 0–9 years had the highest OR. In contrast, those who smoked < 10 CPD for > 35 years had the lowest OR. We did not perform this analysis in female current and past smokers because of the limited sample size, particularly of heavy long smokers.

### Association between the duration of smoking cessation/the age at quitting smoking and obesity in past smokers

In both sexes, there were no significant differences in the prevalence of obesity and age-adjusted OR among groups categorized by duration of smoking cessation (S1 Table). The sub-analysis revealed that male past heavy smokers who had quit within 1 year prior to this examination had a significantly higher OR (age-adjusted OR 1.59, 95% CI; 1.10–2.28, p = 0.01, data not shown) in comparison with never smokers. The other male past smokers showed no significant differences in the OR. We could not analyze this variable for female past smokers because the number who smoked more than 20 CPD was too small (n = 44).

In men, no significant differences in the prevalence of obesity were found among groups with different age at smoking cessation categories (Table 10). However, in women, the elder the age of smoking cessation was, the higher the prevalence of obesity was. In line with this, female past smokers who quit smoking after age 55 had an increased OR for obesity (adjusted OR 1.60, 95% CI 1.05–2.38, p = 0.03).

**Table 10:** Comparison of prevalence of obesity and odds ratio for obesity in past smokers stratified by age of smoking cessation.

				Cochran-Mantel-Haenszel trend test		Multiple logistic regression		
	Age of smoking cessation(year)	n	BMI	Prevalence of obesity (%)	P value†	Odds ratio*	95% CI	P value
**Men**	< 30	420	23.2±3.1	22.9	0.41	0.79	0.62–1.01	0.06
	30–39	566	23.7±3.1	29.9		1.03	0.85–1.26	0.74
	40–54	847	23.6±3.1	28.2		1.04	0.87–1.23	0.69
	55 ≤	758	23.0±3.0	24.7		1	0.83–1.21	0.96
**Women**	<30	214	21.1±2.7	7.9	0.49	0.75	0.44–1.21	0.26
	30–39	151	21.8±3.4	15.9		1.27	0.79–1.95	0.31
	40–54	138	22.2±3.2	21		1.15	0.74–1.71	0.53
	55 ≤	110	23.8±3.6	31.8		1.6	1.05–2.38	0.03

*Prevalence of obesity of never smokers was used as the reference value.

BMI, body mass index

^†^Each P value was calculated by Cochran-Mantel-Haenszel trend test among groups stratified by age of smoking cessation in past smokers.

## Discussion

To our knowledge, this is the first report examining the relationship of smoking and obesity on a large scale in Japanese. There were four main findings in the present study. First, the association between age and the prevalence of obesity varied depending on gender and on smoking status. For example, the association was quadratic in men whereas it was rather linear in female smokers. Second, after adjustment for age, current smokers were less obese compared with never or past smokers in men, whereas no such impact of smoking on obesity was observed in women. Third, the prevalence of obesity tended to increase along with pack-years and CPD, but not with duration of smoking. Also, in male smokers, the risks for obesity were higher in the short-term heavy smokers as compared with the long-term light smokers, even if the pack-years were the same. Fourth, the duration of smoking cessation was not associated with obesity in either sex, whereas female past smokers who quit smoking at a later age tended to be obese.

### Different association between age and the prevalence of obesity in men and women

Significant association between age and the prevalence of obesity has been reported in previous publications [7, 10, 22–24]. However, from the present study, we can indicate two novel findings. First, female never smokers had a 20-year older peak of the quadratic curve compared with male never smokers. Second, relationship of obesity and age was linear in female smokers unlike female never smokers who showed quadratic pattern. These findings may be due to hormonal differences between male and female never smokers, or between smokers and never smokers in female. Studies have shown that estrogens reduce adipose accumulation [26], thus the peak of the quadratic curve of female never smokers might delay until the menopause compared with male never smokers. Alternatively, female smokers are reported to be relatively estrogen-deficient [27], and tend to have earlier menopause [28, 29]. This may cause the accumulation of fat and the linear relationship between age and obesity due to the waning protective effect of estrogen, particularly in menopausal women.

### Current smokers were less obese compared with never or past smokers in men, whereas no such impact of smoking on obesity was observed in women

Age-adjusted BMI and the prevalence of obesity were lower in male current smokers compared with never or past smokers in this study, which is in line with previous reports [6–8, 30]. Alternatively, male past smokers were not more obese compared with never smokers, which differs from previous findings in subjects with Western ethnicities [6–8, 30]. These differences may be due to ethnic differences because a recent survey on obesity among Chinese male adults showed results similar to the present study [10]. In women, however, the present study show neither weight loss among current smoker nor weight gain after smoking cessation. These findings differ from previous reports [6–8, 30]. We can only speculate that these variations are due to difference in study design including the sample size, ethnicity and/or lifestyle factors.

### The prevalence of obesity tended to increase along with pack-years and CPD, but not with duration of smoking

Our results revealed that pack-years were positively associated with indices of obesity in current and past smokers of both genders. This finding is of particular interest in male current smokers, because smoking itself seemed to have an anti-obese impact, whereas conversely, the overall degree of smoking burden seemed to have a pro-obese effect. Kim et al. similarly reported positive dose-dependent associations of pack-years with abdominal and visceral obesity in past/current smokers, although overall obesity measured by BMI did not show such differences among groups with different smoking status [11].

Coexistence of both pro- and anti-obese impacts of smoking on obesity in male smokers deserves discussion. Previous studies have suggested that cigarette smoking itself, in particular nicotine, could decrease body weight by appetite suppression and/or increased energy consumption [5]. In addition, comorbid chronic obstructive pulmonary disease or malignant disease might contribute to decreased BMI. Alternatively, smoking may be associated with obesity through, for example, an unhealthy diet and low physical activity that is often present in heavy smokers [31]. The lesser association of smoking on obesity in women compared with men in the present study might have been due to hormonal or lifestyle differences between men and women in Japan.

In the present study, the number of CPD was positively associated or tended to be associated with indices of obesity in current and past smokers, whereas no consistent trend was observed for duration of smoking in both sexes. These suggest that the positive correlations between pack-years and obesity were primarily derived from CPD. Indeed, such a positive association between CPD and obesity has been reported in past and current smokers [7, 30, 32 –34]. Alternatively, we found significant negative association between smoking duration and obesity in certain subsets of smokers in both sexes. For example, male long-term (> 35 years) smokers had lower age-adjusted ORs for obesity as compared with never smokers. Similarly, Albanes et al. reported that BMI tended to decrease with longer duration of smoking irrespective of the intensity of smoking in the United States population [3]. Interestingly, there may be inter-race differences in the response of obesity to smoking duration because current smokers who had smoked for more than 20 years were more likely to be overweight in a Scottish population [8], which is in contrast to results of our study and that of another study reported from the United States [3]. Also, in the present study, female short-term smokers (< 10 years) had a significantly higher OR for obesity, which may be of social importance considering the wide belief that smoking may be a simple way to lose weight.

Different associations of pack-years, CPD, and the duration of smoking with obesity-related indices mean that use of these indices as risk markers for obesity must be done with caution. For example, 30 pack-years can be calculated in a person with a smoking history of 0.5 pack/day for 60 years as well as in a person with a smoking history of 3 packs/day for 10 years. Importantly, the present study suggested long-term light smokers tended to be less obese and, conversely, short-term heavy smokers tented to represent a more obese subset compared with age-adjusted never smokers. To the best of our knowledge, the present study is the first to concurrently analyze the contribution of CPD and the duration of smoking on obesity, although some studies have separately analyzed the effects of CPD and the duration of smoking [3, 8].

### The duration of smoking cessation was not associated with obesity in either sex, whereas female past smokers who quit smoking at a later age tended to be obese

A series of studies have revealed an increase in body weight after smoking cessation [35–37]. In the present study, male past smokers who smoked ≥ 20 CPD and quit within 1 year prior to this study had a higher OR for obesity compared with never smokers. This result was, at least partly, in accordance with results of prior studies [8, 36, 38, 39]. Alternatively, in women, the older the age of quitting smoking was, the higher the prevalence of obesity was. For example, women who quit smoking after age 55 were more likely to be obese. Interestingly, however, this result is directly opposed to the report from the United States that women younger than 55 years were at higher risk of major weight gain after quitting smoking [37]. These contrasting results might be caused by racial differences, although the true reasons for the different findings cannot be determined because of the observational nature of the studies.

When quitting at younger age, no risk for obesity was found in women in the present study. This was in accordance with results from Scotland [8]. A possible explanation for this is the anti-obese effect of estrogen in younger women as opposed to older, or menopausal, women [5]. This information may be socially important for young female current smokers, who are reluctant to stop smoking because of a fear of gaining weight.

### Limitations

Limitations of our study include, first, that the study was cross-sectional in nature and, therefore, reverse causation cannot be excluded. It is, however, challenging to accurately elucidate temporal relationships between smoking behavior and BMI, and large-scale studies like the present one have been used for the investigation of such relationships. Second, no validation of the smoking data, height, and body weight was done, as all data were self-reported. However, Vartiainen et al. reported a negligible reporting bias regarding smoking in population surveys [39]. Third, though our results were adjusted by age, other potentially confounding factors, such as energy intake, physical activity, alcohol consumption, educational level, and a frequency of failure to quit smoking were not assessed. In addition, no data about pregnancy as a confounding variable were available.

## Conclusions

In conclusion, the present nationwide survey on Japanese adults demonstrated gender-specific associations between age and obesity, varying impacts of CPD and the duration of smoking on obesity. In men, CPD may be a crucial factor in obesity among both current and past smokers. Alternatively, in women, our data did not support any impact of smoking on weight loss, irrespective of the smoking status. Because, in Japan, the smoking rate of women, in particular younger generations, has not decreased overtime [24], Japanese younger women should be provided with information that smoking is not useful for preventing obesity. In addition, younger generations who fear that quitting smoking will lead to weight gain should be informed that scientific data do not support this fear.

## Supporting Information

S1 Table Comparison of prevalence of obesity and odds ratio for obesity in past smokers stratified by the duration of smoking cessation. *Prevalence of obesity of 524 never smokers was used as the reference value. BMI, body mass index.
(PDF)
