# Supplementary material for: Association between Smoking Status and Obesity in a Nationwide Survey of Japanese Adults
Source: PLoS One. 2016 Mar 23;11(3):e0148926. doi: 10.1371/journal.pone.0148926 (PMC4805304; doi:10.1371/journal.pone.0148926)
Supplement: S1 Table — *Prevalence of obesity of 524 never smokers was used as the reference value. BMI, body mass index. (PDF) [file pone.0148926.s001.pdf]

Table S1. Comparison of prevalence of obesity and odds ratio for obesity in past smokers stratified by the duration of smoking cessation

Men

			Cochran-Mantel-Haenszel trend test		Multiple logistic regression		
Duration of smoking cessation (year)	n	BMI	Prevalence of obesity (%)	P value	Odds ratio*	95% CI	P value
0-1	441	23.6± 3.5	30.2		1.17	0.94- 1.71	0.15
2-4	454	23.3± 3.2	26.2		0.92	0.74- 1.15	0.49
5-7	286	23.3± 3.0	25.9	0.14	0.93	0.70- 1.22	0.6
8-10	231	23.2± 3.0	26		0.91	0.66- 1.22	0.53
10 <	1179	23.4± 2.9	25.9		0.96	0.82- 1.11	0.57

Women

			Cochran-Mantel-Haenszel trend test		Multiple logistic regression		
Duration of smoking cessation (year)	n	BMI	Prevalence of obesity (%)	P value	Odds ratio*	95% CI	P value
0-1	122	21.5± 3.2	13.1		1.03	0.58- 1.71	0.92
2-4	137	21.8± 3.5	13.9		1.03	0.61- 1.66	0.91
5-7	106	22.5± 3.4	20.8	0.8	1.57	0.95- 2.50	0.08
8-10	60	22.0± 3.7	16.7		1.09	0.51- 2.08	0.81
10 <	188	22.2± 3.2	20.2		1.18	0.81- 1.68	0.37

*Prevalence of obesity of never smokers was used as the reference value.
BMI, body mass index
